# Correspondence

**Published:** 1881-11

**Authors:** Geo. Watt

**Affiliations:** Xenia, O.


### CORRESPONDENCE.

EDITOR DENTAL REGISTER:—

In reference to the scientific and practical progress of the dental profession, you and I are equally interested. We are of about the same age; and our professional ages and experiences are nearly equal. One was a physician by the time the other was a dentist. In variety of professional experiments we are not far apart. Both have practiced in small and in large towns or cities, both have lectured in dental schools, and both have edited professional journals. And now, what? You say. Ought we not by all means in our power to labor still to advance the cause which has enlisted the energies of the best years of our lives? There we agree again; but how?

Within a few months several of the most useful of our societies will hold their annual meetings, and within striking distance of us, too, if health is found to be reasonably good at the several dates. Some of these societies have almost backslidden. One that used to be noted for the valuable papers read at its meetings, has had few, or no papers read before it for a number of years. Can we wake up the members to do better in this respect? How? Well, talk up the importance of the matter, write to those who can, and who probably will write, if stirred up a little. I shall write to six, for this purpose, between now and New Year's day. Will you do the same? And then, we might tell them to go and do likewise. The oldest, the mother society needs waking up in this respect. She is a good old lady, is old Mrs. Sippi. No doubt you have met her; but let us visit her again. May-be, she likes us.

Then, before calling on her we must go—to the penitentiary, and even further, quite up into the heart of the city where "fair Scioto flows," and attend the Ohio State Society. O! high, oh! but she mustn't remain state-you-quo, even if she is a state society. "State-you-quo," of course means the state or condition in which YOU found her. We must have better essays, clearer discussions, more thorough investigations than lately, or ever before, if we can have them; and we can't have them if we all sit and sleep.

Then the big American, it should not be unwieldy. Especially its standing committees should have their work well in hand, and the members "Alwato" refuse to listen to a report (?) made up of twaddle and nonsense. And it should be totally and thoroughly freed from the influence of spirits, from Gabriel down to the spirits of wine, and especially the spirit of nonsensical gibberish.

So much for the coming meetings. Our colleges and our periodicals should all be improved,—all the journals except the Register and the *Ohio Journal.* They being perfect now, can't be improved. But we must keep them up to their present high standard, and our friends must help us. And of course they will; for even the gods help those who help themselves.

And now, in closing, let me hope that the society meetings referred to will be most excellent in all respects, and that you and I may be there to see. Till then let me be, as ever,

Your friend,

Xenia, O., Oct. 21st, 1881. GEO. WATT.

